# 2-(4-Bromo­phen­yl)-2-methyl-2,3-di­hydro­quinazolin-4(1*H*)-one

**DOI:** 10.1107/S1600536810012584

**Published:** 2010-04-14

**Authors:** Mei-Mei Zhang, Ke Yang, Xiang-Shan Wang

**Affiliations:** a Key Laboratory of Biotechnology for Medical Plants of Jiangsu Province, Xuzhou Normal University, Xuzhou, Jiangsu 221116, People’s Republic of China; b School of Chemistry and Chemical Engineering, Xuzhou Normal University, Xuzhou, Jiangsu 221116, People’s Republic of China

## Abstract

In the title compound, C_15_H_13_BrN_2_O, the pyrimidine ring adopts a skew boat conformation. The amino H atom forms an inter­molecular hydrogen bond with the carbonyl O atom of an adjacent mol­ecule, forming an inversion dimer. Another lone pair of electrons on the same carbonyl O atom acts as acceptor for another N—H⋯O inter­molecular hydrogen bond with a neighbouring mol­ecule, forming chains along the *c* axis.

## Related literature

For biological properties of quinazolinone derivatives, see: Alagarsamy *et al.* (2006 ▶, 2007 ▶); Hwang *et al.* (2008 ▶); Na *et al.* (2008 ▶); Nandy *et al.* (2006 ▶). For related structures, see: Wang *et al.* (2008 ▶); Zhang *et al.* (2009 ▶).
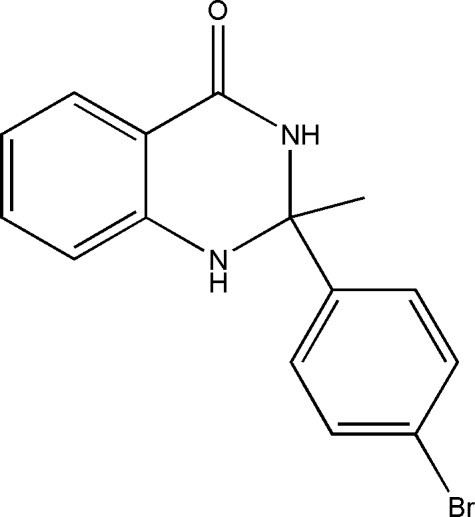


## Experimental

### 

#### Crystal data

C_15_H_13_BrN_2_O
*M* *_r_* = 317.18
Monoclinic, 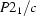

*a* = 12.2106 (3) Å
*b* = 9.0507 (2) Å
*c* = 12.4046 (3) Å
β = 101.719 (1)°
*V* = 1342.31 (5) Å^3^
*Z* = 4
Mo *K*α radiation
μ = 3.06 mm^−1^
*T* = 296 K
0.39 × 0.31 × 0.07 mm

#### Data collection

Bruker SMART CCD area-detector diffractometer
Absorption correction: multi-scan (*SADABS*; Sheldrick, 2001 ▶) *T*_min_ = 0.343, *T*_max_ = 0.801
16905 measured reflections
2369 independent reflections
2068 reflections with *I* > 2σ(*I*)
*R*_int_ = 0.025

#### Refinement

*R*[*F* ^2^ > 2σ(*F* ^2^)] = 0.034
*wR*(*F* ^2^) = 0.087
*S* = 1.05
2369 reflections
181 parameters
2 restraints
H atoms treated by a mixture of independent and constrained refinement
Δρ_max_ = 0.89 e Å^−3^
Δρ_min_ = −0.86 e Å^−3^

### 

Data collection: *SMART* (Bruker, 2001 ▶); cell refinement: *SMART*; data reduction: *SAINT* (Bruker, 2001 ▶); program(s) used to solve structure: *SHELXS97* (Sheldrick, 2008 ▶); program(s) used to refine structure: *SHELXL97* (Sheldrick, 2008 ▶); molecular graphics: *SHELXTL* (Sheldrick, 2008 ▶); software used to prepare material for publication: *SHELXTL*.

## Supplementary Material

Crystal structure: contains datablocks global, I. DOI: 10.1107/S1600536810012584/pv2270sup1.cif

Structure factors: contains datablocks I. DOI: 10.1107/S1600536810012584/pv2270Isup2.hkl

Additional supplementary materials:  crystallographic information; 3D view; checkCIF report

## Figures and Tables

**Table 1:** Hydrogen-bond geometry (Å, °)

*D*—H⋯*A*	*D*—H	H⋯*A*	*D*⋯*A*	*D*—H⋯*A*
N1—H1⋯O1^i^	0.85 (1)	2.08 (1)	2.932 (3)	179 (3)
N2—H2⋯O1^ii^	0.85 (1)	2.04 (1)	2.870 (3)	164 (3)

Symmetry codes: (i) 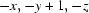; (ii) 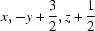.

**Fig. 1.**
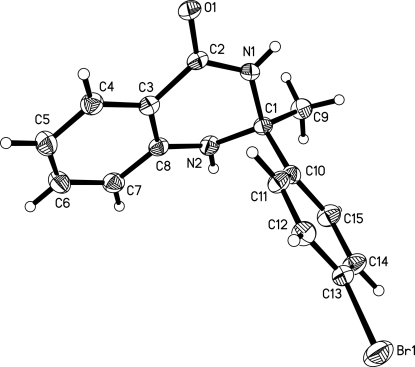
The molecular structure drawing for (I) showing 30% probability of displacement ellipsoids and the atom-numbering scheme.

**Fig. 2.**
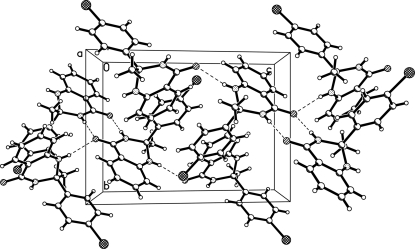
The molecular packing diagram of (I).

## supplementary crystallographic information

### Comment

The synthesis of quinazolinone derivatives has been the focus of great interest, because it was reported that its derivatives possessed a broad spectrum of biological properties. Some of these activities include antidepressant (Na *et al.*, 2008), anticancer (Hwang *et al.*, 2008), anti-inflammatory (Alagarsamy, *et al.*, 2007), antibacterial (Alagarsamy *et al.*, 2006), and antitubercular activity (Nandy *et al.*, 2006). The title compound may be used as a new precursor for obtaining bioactive molecules. We report here the crystal structure of the title compound, (I).

In the title molecule the pyrimidine ring of the quinazolinone moiety is slightly distorted and adopts a skew conformation (Fig. 1). The atoms C1 and N1 deviate from the basal plane defined by the atoms C2/C3/C8/N2 by 0.631 (4) and 0.222 (4) Å, respectively. Similar structures were observed in the structures of 2-(4-chloroanilino)-3-(2-hydroxyethyl)-quinazolin-4(3*H*)-one (Wang *et al.*, 2008) and 3-(2-hydroxyethyl)-2-(*p*-tolylamino)-quinazolin-4(3*H*)-one (Zhang *et al.*, 2009). In (I), the basal plane of the pyrimidine ring is nearly parallel to the phenyl ring C3/C4/C5/C6/C7/C8, forming a dihedral angle of 4.5 (2)°, and is nearly perpendicular to another 4-bromophenyl ring, forming a dihedral angle of 82.2 (1)°.

Intermolecular N1—H1···O1 hydrogen bonds (Table 1) are formed between the amino and carbonyl groups, and link the moleclues forming dimers (Fig. 2). Another intermolecular N2—H2···O1 hydrogen bond links the neighbouring molecules forming polymeric chains along the *c*-axis.

### Experimental

The title compound was prepared by the reaction of 2-aminobenzamide (0.272 g, 2 mmol) and 4'-bromoacetophenone (0.398 g, 2 mmol) in the presence of iodine (0.026 g) in tetrahydrofuran at 323 K for 6 h (yield 86%, m.p. 494–496 K). Crystals of (I) suitable for X-ray diffraction were obtained by slow evaporation of a dimethylformamide solution.

### Refinement

The H atoms bonded to C atoms were included at geometrically idealized positions and refined in riding-model approximation with C—H = 0.93 and 0.96 Å, for aryl and methyl H atoms, respectively; the H atoms bonded to N atoms were allowed to refine. The *U*_iso_(H) were allowed at 1.2*U*_eq_(parent atoms). The final difference map was essentially featurless with the residual electron density located in the close proximity of the Br1 atom.

### Crystal data

**Table:**

C_15_H_13_BrN_2_O	*F*(000) = 640
*M**_r_* = 317.18	*D*_x_ = 1.570 Mg m^−^^3^
Monoclinic, *P*2_1_/*c*	Melting point = 494–496 K
Hall symbol: -P 2ybc	Mo *K*α radiation, λ = 0.71073 Å
*a* = 12.2106 (3) Å	Cell parameters from 7144 reflections
*b* = 9.0507 (2) Å	θ = 2.8–27.1°
*c* = 12.4046 (3) Å	µ = 3.06 mm^−^^1^
β = 101.719 (1)°	*T* = 296 K
*V* = 1342.31 (5) Å^3^	Block, colourless
*Z* = 4	0.39 × 0.31 × 0.07 mm

### Data collection

**Table:**

Bruker SMART CCD area-detector diffractometer	2369 independent reflections
Radiation source: fine-focus sealed tube	2068 reflections with *I* > 2σ(*I*)
graphite	*R*_int_ = 0.025
φ and ω scans	θ_max_ = 25.0°, θ_min_ = 1.7°
Absorption correction: multi-scan (*SADABS*; Sheldrick, 2001)	*h* = −14→14
*T*_min_ = 0.343, *T*_max_ = 0.801	*k* = −10→10
16905 measured reflections	*l* = −14→14

### Refinement

**Table:**

Refinement on *F*^2^	Primary atom site location: structure-invariant direct methods
Least-squares matrix: full	Secondary atom site location: difference Fourier map
*R*[*F*^2^ > 2σ(*F*^2^)] = 0.034	Hydrogen site location: inferred from neighbouring sites
*wR*(*F*^2^) = 0.087	H atoms treated by a mixture of independent and constrained refinement
*S* = 1.05	*w* = 1/[σ^2^(*F*_o_^2^) + (0.0402*P*)^2^ + 1.4299*P*] where *P* = (*F*_o_^2^ + 2*F*_c_^2^)/3
2369 reflections	(Δ/σ)_max_ < 0.001
181 parameters	Δρ_max_ = 0.89 e Å^−^^3^
2 restraints	Δρ_min_ = −0.86 e Å^−^^3^

### Special details

**Table:**

Geometry. All esds (except the esd in the dihedral angle between two l.s. planes) are estimated using the full covariance matrix. The cell esds are taken into account individually in the estimation of esds in distances, angles and torsion angles; correlations between esds in cell parameters are only used when they are defined by crystal symmetry. An approximate (isotropic) treatment of cell esds is used for estimating esds involving l.s. planes.
Refinement. Refinement of *F*^2^ against ALL reflections. The weighted *R*-factor wR and goodness of fit *S* are based on *F*^2^, conventional *R*-factors *R* are based on *F*, with *F* set to zero for negative *F*^2^. The threshold expression of *F*^2^ > σ(*F*^2^) is used only for calculating *R*-factors(gt) etc. and is not relevant to the choice of reflections for refinement. *R*-factors based on *F*^2^ are statistically about twice as large as those based on *F*, and *R*- factors based on ALL data will be even larger.

### Fractional atomic coordinates and isotropic or equivalent isotropic displacement parameters (Å^2^)

**Table:**

	*x*	*y*	*z*	*U*_iso_*/*U*_eq_	
Br1	0.39200 (3)	0.15530 (4)	0.53610 (3)	0.06231 (16)	
O1	0.12331 (16)	0.5903 (2)	−0.02983 (14)	0.0438 (5)	
N2	0.14117 (19)	0.7611 (2)	0.27005 (18)	0.0356 (5)	
C2	0.1400 (2)	0.6296 (3)	0.0687 (2)	0.0331 (6)	
C10	0.1575 (2)	0.4972 (3)	0.3192 (2)	0.0305 (5)	
N1	0.07053 (18)	0.5872 (3)	0.13329 (17)	0.0341 (5)	
C15	0.1462 (2)	0.4648 (3)	0.4258 (2)	0.0423 (6)	
H15A	0.0918	0.5136	0.4550	0.051*	
C8	0.2278 (2)	0.7955 (3)	0.2193 (2)	0.0339 (6)	
C7	0.3078 (2)	0.9022 (3)	0.2615 (2)	0.0430 (7)	
H7A	0.3047	0.9501	0.3271	0.052*	
C11	0.2387 (2)	0.4209 (3)	0.2788 (2)	0.0371 (6)	
H11A	0.2471	0.4391	0.2071	0.045*	
C3	0.2318 (2)	0.7284 (3)	0.1177 (2)	0.0342 (6)	
C9	−0.0294 (2)	0.6307 (3)	0.2795 (2)	0.0418 (6)	
H9A	−0.0752	0.6953	0.2278	0.050*	
H9B	−0.0224	0.6699	0.3525	0.050*	
H9C	−0.0633	0.5346	0.2760	0.050*	
C1	0.0863 (2)	0.6182 (3)	0.2514 (2)	0.0312 (5)	
C13	0.2944 (2)	0.2903 (3)	0.4473 (2)	0.0396 (6)	
C14	0.2138 (3)	0.3620 (3)	0.4894 (2)	0.0462 (7)	
H14A	0.2046	0.3415	0.5605	0.055*	
C4	0.3171 (2)	0.7652 (3)	0.0637 (2)	0.0417 (6)	
H4A	0.3202	0.7199	−0.0030	0.050*	
C5	0.3972 (2)	0.8676 (4)	0.1070 (3)	0.0494 (7)	
H5A	0.4546	0.8906	0.0707	0.059*	
C12	0.3077 (2)	0.3185 (3)	0.3416 (2)	0.0416 (6)	
H12A	0.3623	0.2693	0.3129	0.050*	
C6	0.3913 (3)	0.9364 (3)	0.2060 (3)	0.0500 (7)	
H6A	0.4447	1.0067	0.2352	0.060*	
H2	0.146 (2)	0.794 (3)	0.3350 (12)	0.042 (8)*	
H1	0.0147 (16)	0.535 (3)	0.103 (2)	0.035 (7)*	

### Atomic displacement parameters (Å^2^)

**Table:**

	*U*^11^	*U*^22^	*U*^33^	*U*^12^	*U*^13^	*U*^23^
Br1	0.0575 (2)	0.0674 (3)	0.0598 (2)	0.01474 (16)	0.00690 (16)	0.02754 (17)
O1	0.0550 (12)	0.0520 (12)	0.0237 (10)	−0.0116 (9)	0.0066 (8)	−0.0021 (8)
N2	0.0477 (13)	0.0326 (11)	0.0280 (12)	−0.0021 (10)	0.0111 (10)	−0.0048 (9)
C2	0.0381 (14)	0.0331 (13)	0.0269 (13)	0.0041 (11)	0.0038 (10)	0.0031 (10)
C10	0.0313 (12)	0.0325 (13)	0.0277 (13)	−0.0051 (10)	0.0064 (10)	−0.0031 (10)
N1	0.0361 (12)	0.0392 (12)	0.0257 (11)	−0.0056 (10)	0.0030 (9)	−0.0014 (9)
C15	0.0455 (16)	0.0506 (17)	0.0340 (15)	0.0063 (13)	0.0156 (12)	0.0007 (13)
C8	0.0374 (14)	0.0318 (13)	0.0311 (14)	0.0028 (11)	0.0036 (11)	0.0024 (11)
C7	0.0515 (17)	0.0393 (15)	0.0359 (15)	−0.0041 (13)	0.0030 (12)	−0.0031 (12)
C11	0.0417 (14)	0.0418 (15)	0.0301 (14)	0.0035 (12)	0.0129 (11)	0.0039 (11)
C3	0.0374 (14)	0.0353 (13)	0.0294 (13)	0.0018 (11)	0.0057 (11)	0.0016 (11)
C9	0.0376 (14)	0.0474 (16)	0.0416 (16)	0.0047 (12)	0.0111 (12)	0.0009 (13)
C1	0.0346 (13)	0.0338 (13)	0.0257 (13)	−0.0010 (10)	0.0075 (10)	−0.0022 (10)
C13	0.0366 (14)	0.0401 (15)	0.0394 (15)	−0.0012 (12)	0.0013 (11)	0.0088 (12)
C14	0.0519 (17)	0.0572 (18)	0.0307 (15)	0.0038 (14)	0.0116 (13)	0.0083 (13)
C4	0.0403 (15)	0.0485 (16)	0.0370 (15)	0.0016 (13)	0.0100 (12)	0.0010 (12)
C5	0.0365 (15)	0.0568 (19)	0.056 (2)	−0.0031 (13)	0.0116 (13)	0.0052 (15)
C12	0.0381 (15)	0.0444 (15)	0.0438 (16)	0.0054 (12)	0.0120 (12)	0.0021 (13)
C6	0.0428 (16)	0.0477 (17)	0.0556 (19)	−0.0108 (13)	0.0011 (14)	0.0004 (14)

### Geometric parameters (Å, °)

**Table:**

Br1—C13	1.896 (3)	C7—H7A	0.9300
O1—C2	1.249 (3)	C11—C12	1.382 (4)
N2—C8	1.372 (3)	C11—H11A	0.9300
N2—C1	1.453 (3)	C3—C4	1.389 (4)
N2—H2	0.851 (10)	C9—C1	1.526 (4)
C2—N1	1.336 (3)	C9—H9A	0.9600
C2—C3	1.466 (4)	C9—H9B	0.9600
C10—C11	1.384 (4)	C9—H9C	0.9600
C10—C15	1.388 (4)	C13—C14	1.368 (4)
C10—C1	1.538 (3)	C13—C12	1.378 (4)
N1—C1	1.466 (3)	C14—H14A	0.9300
N1—H1	0.854 (10)	C4—C5	1.375 (4)
C15—C14	1.381 (4)	C4—H4A	0.9300
C15—H15A	0.9300	C5—C6	1.391 (4)
C8—C7	1.397 (4)	C5—H5A	0.9300
C8—C3	1.409 (4)	C12—H12A	0.9300
C7—C6	1.376 (4)	C6—H6A	0.9300
			
C8—N2—C1	120.2 (2)	H9A—C9—H9B	109.5
C8—N2—H2	116 (2)	C1—C9—H9C	109.5
C1—N2—H2	114 (2)	H9A—C9—H9C	109.5
O1—C2—N1	120.5 (2)	H9B—C9—H9C	109.5
O1—C2—C3	122.6 (2)	N2—C1—N1	107.0 (2)
N1—C2—C3	116.8 (2)	N2—C1—C9	108.4 (2)
C11—C10—C15	117.2 (2)	N1—C1—C9	107.6 (2)
C11—C10—C1	121.6 (2)	N2—C1—C10	110.9 (2)
C15—C10—C1	121.1 (2)	N1—C1—C10	110.8 (2)
C2—N1—C1	125.0 (2)	C9—C1—C10	112.0 (2)
C2—N1—H1	116.3 (19)	C14—C13—C12	120.6 (3)
C1—N1—H1	118.5 (19)	C14—C13—Br1	120.0 (2)
C14—C15—C10	121.6 (3)	C12—C13—Br1	119.4 (2)
C14—C15—H15A	119.2	C13—C14—C15	119.6 (3)
C10—C15—H15A	119.2	C13—C14—H14A	120.2
N2—C8—C7	122.1 (2)	C15—C14—H14A	120.2
N2—C8—C3	118.9 (2)	C5—C4—C3	121.1 (3)
C7—C8—C3	118.9 (2)	C5—C4—H4A	119.5
C6—C7—C8	120.1 (3)	C3—C4—H4A	119.5
C6—C7—H7A	120.0	C4—C5—C6	119.1 (3)
C8—C7—H7A	120.0	C4—C5—H5A	120.4
C12—C11—C10	122.0 (2)	C6—C5—H5A	120.4
C12—C11—H11A	119.0	C13—C12—C11	119.0 (3)
C10—C11—H11A	119.0	C13—C12—H12A	120.5
C4—C3—C8	119.6 (2)	C11—C12—H12A	120.5
C4—C3—C2	122.1 (2)	C7—C6—C5	121.1 (3)
C8—C3—C2	118.1 (2)	C7—C6—H6A	119.4
C1—C9—H9A	109.5	C5—C6—H6A	119.4
C1—C9—H9B	109.5		
			
O1—C2—N1—C1	175.6 (2)	C2—N1—C1—N2	33.1 (3)
C3—C2—N1—C1	−7.2 (4)	C2—N1—C1—C9	149.4 (2)
C11—C10—C15—C14	0.6 (4)	C2—N1—C1—C10	−87.8 (3)
C1—C10—C15—C14	−176.5 (3)	C11—C10—C1—N2	−88.1 (3)
C1—N2—C8—C7	−156.5 (2)	C15—C10—C1—N2	88.9 (3)
C1—N2—C8—C3	27.8 (4)	C11—C10—C1—N1	30.5 (3)
N2—C8—C7—C6	−178.0 (3)	C15—C10—C1—N1	−152.5 (2)
C3—C8—C7—C6	−2.4 (4)	C11—C10—C1—C9	150.7 (2)
C15—C10—C11—C12	−1.2 (4)	C15—C10—C1—C9	−32.4 (3)
C1—C10—C11—C12	175.9 (2)	C12—C13—C14—C15	−0.8 (5)
N2—C8—C3—C4	178.1 (2)	Br1—C13—C14—C15	177.2 (2)
C7—C8—C3—C4	2.4 (4)	C10—C15—C14—C13	0.4 (5)
N2—C8—C3—C2	2.7 (4)	C8—C3—C4—C5	−0.8 (4)
C7—C8—C3—C2	−173.1 (2)	C2—C3—C4—C5	174.5 (3)
O1—C2—C3—C4	−11.0 (4)	C3—C4—C5—C6	−0.8 (4)
N1—C2—C3—C4	171.9 (2)	C14—C13—C12—C11	0.3 (4)
O1—C2—C3—C8	164.4 (2)	Br1—C13—C12—C11	−177.8 (2)
N1—C2—C3—C8	−12.8 (3)	C10—C11—C12—C13	0.7 (4)
C8—N2—C1—N1	−43.0 (3)	C8—C7—C6—C5	0.8 (5)
C8—N2—C1—C9	−158.8 (2)	C4—C5—C6—C7	0.8 (5)
C8—N2—C1—C10	77.9 (3)		

### Hydrogen-bond geometry (Å, °)

**Table:**

*D*—H···*A*	*D*—H	H···*A*	*D*···*A*	*D*—H···*A*
N1—H1···O1^i^	0.85 (1)	2.08 (1)	2.932 (3)	179 (3)
N2—H2···O1^ii^	0.85 (1)	2.04 (1)	2.870 (3)	164 (3)

Symmetry codes: (i) −*x*, −*y*+1, −*z*; (ii) *x*, −*y*+3/2, *z*+1/2.
